# Prognostic indicators for hospitalization and ICU admission in people with multiple sclerosis and COVID-19: an analysis of the COVID-19 in MS global data sharing initiative dataset

**DOI:** 10.1097/MS9.0000000000001676

**Published:** 2024-01-03

**Authors:** Maria A. Garcia-Dominguez, Vincent Kipkorir, Bahadar S. Srichawla

**Affiliations:** a Department of Neurology, University of Massachusetts Chan Medical School, MA, USA; b Department of Medicine, University of Nairobi, Nairobi, Kenya

**Keywords:** COVID-19, disease-modifying therapy, hospitalization, intensive care unit, multiple sclerosis, people with multiple sclerosis, SARS-CoV-2

## Abstract

**Objectives:**

To analyze the symptoms and severity of coronavirus disease 2019 (COVID-19) in people with multiple sclerosis (pwMS) on disease-modifying therapies using data from the COVID-19 in multiple sclerosis (MS) Global Data Sharing Initiative dataset.

**Methods:**

The open-access COVID-19 in MS Global Data Sharing Initiative dataset was obtained through credentialed access using PhysioNet. The variables analyzed included BMI, symptoms of COVID-19, age, current use of disease-modifying therapy (DMT), efficacy of DMT, comorbidities, hospitalization status, and type of MS. A linear regression analysis was completed. Data analysis and visualization were completed using STATA *v15*, R-Studio *v1.1.447*, Python *v3.8,* and its associated libraries, including NumPy, Pandas, and Matplotlib.

**Results:**

A total of 1141 participants were included in the analysis. 904 women and 237 men were diagnosed with MS. Among the pwMS included in the study; 208 (19.54%) had a suspected infection with COVID-19 and only 49 (5.25%) were confirmed. Any COVID-19 symptom was present in 360 individuals. The commonly reported DMT agents included dimethyl fumarate (12.71%) and fingolimod (10.17%). 101 in total (8.85%) reported not using any DMT. Factors associated with hospitalization and/or admission to the ICU included having any comorbidity (*P=*0.01), neuromuscular disorder (*P=*0.046), hypertension (*P=*0.005), chronic kidney disease (*P<*0.001), and immunodeficiency (*P=*0.003). The type of MS, the duration of the disease, and high-efficacy DMT therapy did not have a statistically significant influence on hospitalization.

**Conclusion:**

This study underscores the importance of comorbidities, especially neuromuscular disorders, hypertension, chronic kidney disease, and immunodeficiencies, as possible prognostic indicators for worse outcomes of COVID-19 in pwMS. On the contrary, the type of MS, the duration of the disease, and the efficacy of disease-modifying therapy did not significantly affect the severity of the symptoms of COVID-19 in this cohort.

## Introduction

Highlights

- The study analyzed symptoms and severity of coronavirus disease 2019 (COVID-19) in people with multiple sclerosis using data from the COVID-19 in multiple sclerosis (MS) Global Data Sharing Initiative dataset.
- The study included 1141 participants, out of which 208 had suspected COVID-19 infections, and only 49 were confirmed. Commonly used disease-modifying therapies were dimethyl fumarate and fingolimod.
- Factors significantly associated with hospitalization or ICU admission included comorbidities like neuromuscular disorders, hypertension, chronic kidney disease, and immunodeficiencies.
- The type of MS, duration of the disease, and the efficacy of disease-modifying therapies did not have a statistically significant impact on COVID-19 severity or hospitalization rates among the study participants.
- The study concluded that comorbidities are key indicators for worse COVID-19 outcomes in people with multiple sclerosis, while factors specific to MS, such as type or treatment efficacy, did not significantly affect severity.

The emergence of the novel coronavirus disease 2019 (COVID-19), caused by the severe acute respiratory syndrome coronavirus 2 (SARS-CoV-2), has resulted in a global pandemic with significant morbidity and mortality^1^. The pathophysiology of SARS-CoV-2 is believed to involve virus utilization of angiotensin-converting enzyme 2 (ACE2) as a cell receptor for viral entry^2^. As the pandemic has evolved, researchers have aimed to identify specific populations that may be at increased risk of adverse outcomes from the virus. One of such populations of interest is people with multiple sclerosis (pwMS)^3^.

Multiple sclerosis (MS) is a chronic autoimmune disease characterized by inflammation, demyelination, and neurodegeneration in the central nervous system^4^. MS patients often require immunosuppressive or immunomodulatory treatments, which can alter their susceptibility to infections, including viruses such as SARS-CoV-2. Disease-modifying therapies (DMTs) in MS aim to alter the course of the disease, reducing the frequency and severity of clinical relapses and slowing the progression of disability. These therapies primarily target the immune system to inhibit its aberrant activity against the central nervous system^5^. Initial concerns early on during the COVID-19 pandemic arose that MS patients might be at higher risk of severe COVID-19 outcomes due to their underlying autoimmune disease and the DMTs they receive^6^. This is due to the immunosuppressive effects of DMTs. However, the exact relationship between MS and COVID-19 outcomes remains to be fully elucidated. Some studies suggest that the overall risk for people with MS contracting COVID-19 does not appear to increase, but once infected, the course and outcomes of the disease can vary^7^.

With the availability of large-scale open-access databases, such as the MS Global Data Sharing Initiative dataset from PhysioNet, a comprehensive analysis can provide valuable insights into the interplay between MS and COVID-19. PhysioNet is an open-access resource dedicated to the collaborative development and sharing of biomedical datasets and software. Established by the National Institute of Biomedical Imaging and Bioengineering (NIBIB) and the National Institute of General Medical Sciences (NIGMS), it serves as a platform for researchers worldwide to share and study complex biomedical and physiological data^8^.

**Figure 1:**
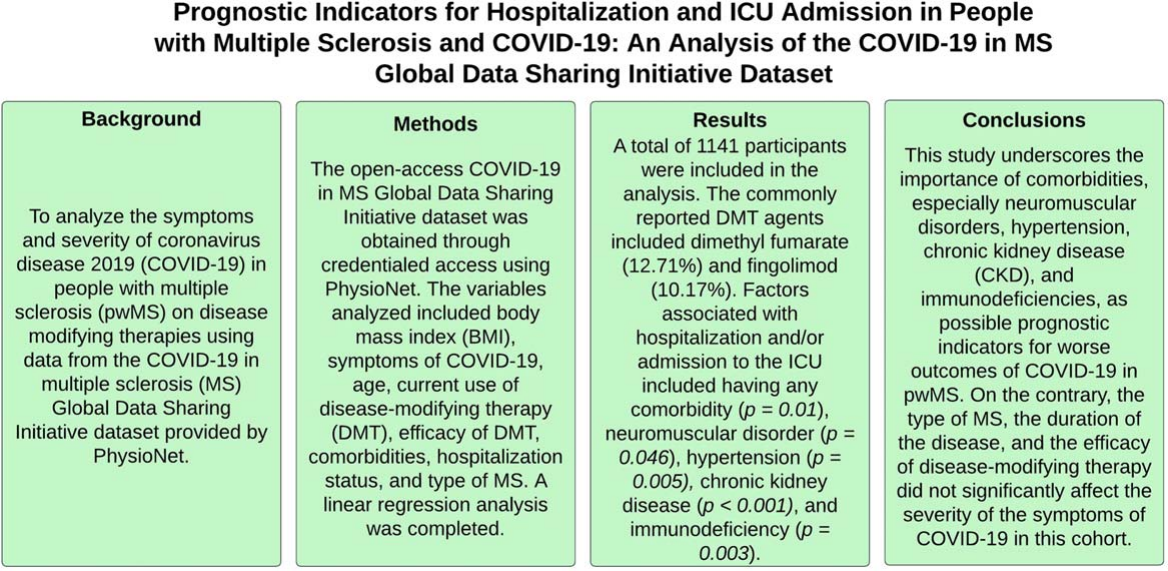
Graphical abstract depicting study rationale, methodology, and key findings and conclusions.

In this study, our objective was to explore the effects of immunomodulatory therapies on COVID-19 in pwMS using an open-access database from the MS Global Data Sharing Initiative, offering a robust perspective on the implications of this global health crisis and providing guidance on future viral pandemics in pwMS (Fig. 1)^9^. Additionally, this study aims to fill the gap in understanding the relationship between MS, DMTs, and COVID-19 outcomes.

## Methods

### Data source and characteristics

This open-access dataset was obtained from PhysioNet (https://physionet.org/content/patient-level-data-covid-ms/1.0.0/)^9^. The data collection methodology has been previously described by *Peeters et al*.^10^. This dataset was chosen because it is the most comprehensive open-access dataset on pwMS and COVID-19. Strengths of this database include it is large sample size, comorbidity data, and tracking of DMTs. Limitations include the lack of expanded disability status scale (EDSS) data, and missing symptomatology data for most patients. Briefly, the dataset was collected through a data entry tool that allowed clinicians, pwMS, or their healthcare agents to enter data directly into a central platform of the COVID-19 and MS Global Data Sharing Initiative. The initial proposal for data collection was published in July of 2020 and the tool was taken down on 2 February 2022. Numerous variables (*n*=47) were recorded, including patient demographics, COVID-19 symptoms, hospitalization, admission to the ICU, DMTs, and EDSS. However, many of these variables had incomplete data and therefore were not included in the analysis presented here. This includes the EDSS as the score was missing for 54.16% of the database. Researchers who accessed the anonymized database completed the required courses to obtain credentialed access. The requirement of individual patient consent was waived because the project did not directly access patient records. The diagnosis of COVID-19, whether ‘suspected’ or ‘confirmed’, was based on standard clinical and laboratory criteria prevalent during the data collection period. It is important to note that ‘suspected’ cases might not have undergone confirmatory polymerase chain reaction testing, and hence, there may be inherent variability in this classification. The following COVID-19 outcomes are defined. Level 0: If the person has COVID-19 but has not been hospitalized. Level 1: The person has COVID-19 and has been hospitalized. Level 2: The person has COVID-19, has been hospitalized, has been in the ICU, and/or was in a ventilation facility. No individuals within this dataset died from COVID-19. The defined outcomes (Levels 0, 1, and 2) are standardized and provide a clear gradation of disease severity. Age groups were predefined by the developers of the database and defined as the following: 1: if the age range is between 18 and ≤50. 2: if the age range is between 51 and ≤70. Complete details regarding the predefined groups are available at https://physionet.org/content/patient-level-data-covid-ms/1.0.0/.

DMTs for MS are categorized according to their efficacy in reducing relapse rates, preventing disability progression, and managing MRI activity (new or enlarging lesions). Their classification into low, medium, or high-efficacy is often based on a combination of clinical trial data, real-world evidence, and their mechanism of action. Low efficacy included glatiramer, interferon, and teriflunomide. Medium efficacy included fingolimod, cladribine, and dimethyl fumarate; high-efficacy included ocrelizumab, alemtuzumab, natalizumab, and rituximab. A category for ‘other’ was included if not listed. This study was completed according to the Strengthening the Reporting of Cohort Studies in Surgery (STROCSS) criteria^11^.

### Statistical analysis

Descriptive statistics were used to summarize clinical data. Categorical variables were summarized with frequencies. Categorical variables were compared between category etiology groups using Fisher’s exact test. Association between the COVID-19 outcomes and the following variables: The type of MS, duration of the disease, admission to the ICU, being overweight, age, currently on a DMT, DMT efficacy, never treated with a DMT, and comorbidities were evaluated using a univariate linear regression model. Prior to analysis, we assessed the distribution of variables. For those not adhering to a normal distribution, we applied appropriate transformations or nonparametric tests. Our primary objective was to model and predict the outcome based on a range of factors. Regression allows for a more nuanced understanding of how each variable contributes to the outcome, adjusting for other variables in the model. Prior to the regression analysis, potential confounders were identified based on their known or suspected association with both the exposure (independent variable) and the outcome (dependent variable). For instance, variables like age, sex, and disease duration were considered as potential confounders due to their established associations with both the exposure of interest and the outcome.

It is worth noting that the primary assumption we examined in linear regression is the linearity of the relationship between the variables. The distribution of individual predictor variables is less of a concern than ensuring the residuals from the model are normally distributed. We verified the normality of the residuals by examining residual plots, which did not show significant deviations from normality. For the statistical inference in our regression models, we employed the *t*-test to evaluate the significance of individual predictors in simple regression settings and coefficients in multiple regression scenarios. The overall fit of the model was gauged using the *F*-test. It is pertinent to mention that the linear regression approach inherently takes these tests into account, ensuring the robustness and validity of our findings. For each regression coefficient obtained, a 95% CI was calculated to estimate the range within which the true population parameter is expected to fall with 95% certainty. The CIs were derived based on the standard errors of the regression coefficients, which reflect the variability of the estimates.

All analyses were performed using STATA *ver 15*, R-Studio *ver 1.1.447*, Python *ver 3.8* including the statistical packages Pandas *ver 1.3.2* and NumPy 1.19. Data visualization was completed using the Python library Matplotlib *ver 3.4.3*. A *P*-value of less than 0.05 was considered statistically significant.

### Bias assessment

Given that our dataset was obtained from the MS Global Data Sharing Initiative which allowed for direct data entry by clinicians, pwMS, or their healthcare agents, there is a potential for selection bias. To address this, we ensured that our analysis accounted for the entirety of the dataset rather than a subset. This mitigates the chances of bias arising from the exclusion of specific patient groups. Since some of the data (like symptoms or comorbidities) might have been self-reported by patients or their caregivers, there is a potential for recall bias. However, the immediate and centralized nature of the data collection platform minimizes the time gap between experience and reporting, reducing this bias. We acknowledge that there might be some degree of misclassification, especially in the ‘suspected’ cases of COVID-19. In our analysis, we ensured to differentiate between ‘suspected’ and ‘confirmed’ cases to minimize the impact of this bias on our results. The data collection tool was decommissioned in early 2022, and there is potential bias arising from the period of data collection, especially given the evolving nature of the pandemic and the emergence of new SARS-CoV-2 variants. We have made this limitation clear in our discussion to ensure transparency.

## Results

A total of 1141 participants, 904 women and 237 men, were diagnosed with MS. Eight hundred eighty-three were in age category 1 (include age range) and 258 in age category two (include age range). Among the MS patients included in the study, 858 had no suspected COVID-19, 208 patients had a suspected COVID-19 infection, and 49 were confirmed. A total of 360 participants reported any symptoms of COVID-19. One hundred one reported chills, 196 dry coughs, 239 fatigue, 141 fever, 88 loss of smell and taste, 156 nasal congestion, 186 pain, 14 pneumonia, 103 shortness of breath, and 169 sore throat.

**Figure 2:**
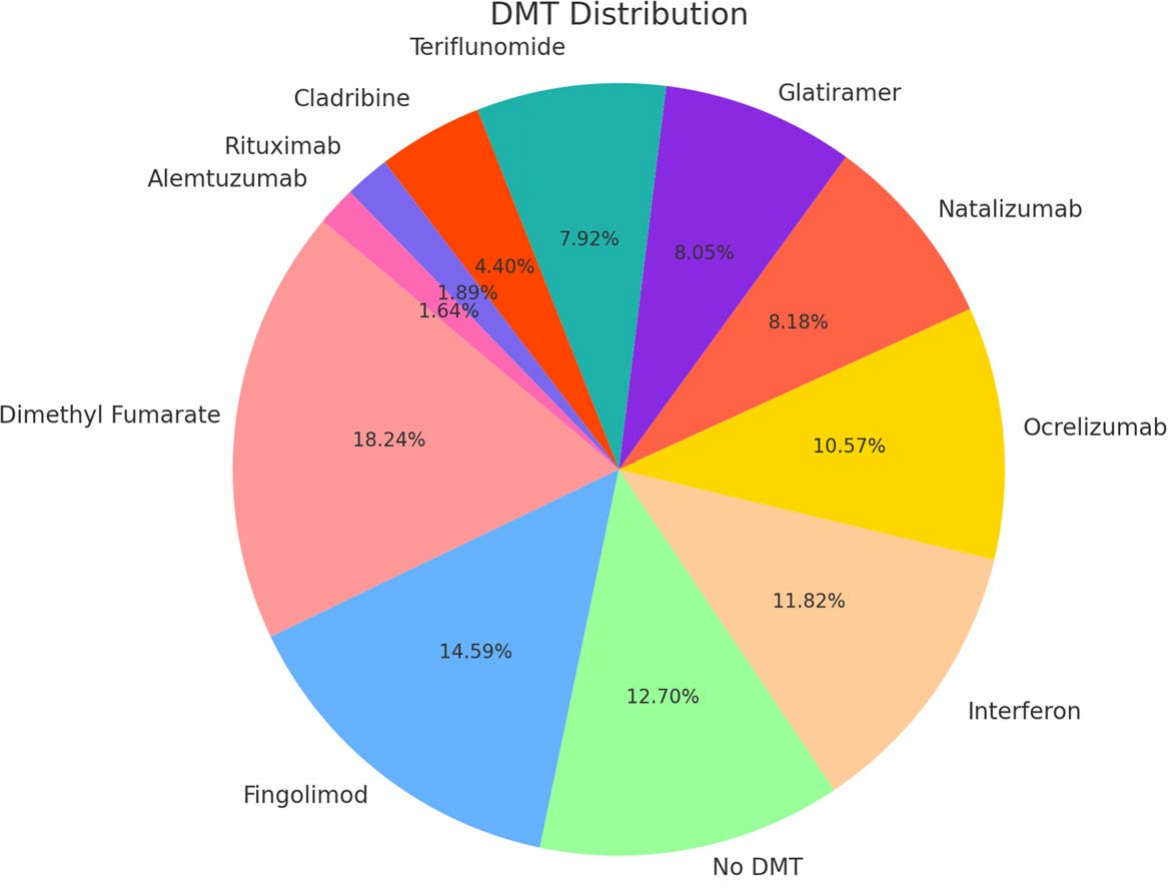
Distribution of disease-modifying therapies (DMTs) among people with multiple sclerosis.

The DMT most frequently reported was an unspecified drug not listed in the dataset, with 185 occurrences (16.21%). This was closely followed by dimethyl fumarate with 145 instances (12.71%) and fingolimod with 116 occurrences (10.17%). A notable number of participants, 101 in total (8.85%), reported not using any DMT. Other therapies such as interferon, reported by 94 participants (8.24%), ocrelizumab with 84 occurrences (7.36%), and natalizumab, reported by 65 individuals (5.70%), were also substantially represented in the data. The less-used DMT included glatiramer with 64 occurrences (5.61%), teriflunomide with 63 instances (5.52%), and cladribine, chosen by 35 participants (3.07%). The least represented DMTs in the study were rituximab with 15 occurrences (1.31%) and alemtuzumab with 13 instances (1.14%). Additionally, data was not available for 161 individuals, which were excluded from percentage calculations (Fig. 2).

Four patients were admitted to the ICU and required ventilation. Eight hundred eighty were currently on DMT, and 48 were on corticosteroids. A total of 148 individuals reported having at least one comorbidity. Factors associated with worse COVID-19-related outcomes include admission to the ICU [B 7.33; *P*<0.0001 (95% CI regression coefficient 5.18–9.48)], having any comorbidity [B 1.02; *P*=0.017 (95% CI regression coefficient 0.64–2.60)], hypertension [B 1.11; *P*=0.005 (95% CI regression coefficient 0.11–2.11)], chronic kidney disease [B 1.62; *P*<0.0001 (95% CI regression coefficient −0.53–3.77)] neuromuscular disorder [B 1.07; *P*=0.046 (95% CI regression coefficient 0.04–2.10)], and immunodeficiency (B 1.09; *P*=0.03). The type of MS (e.g. relapsing-remitting, primary progressive, etc.), duration of the disease, and the DMT efficacy group did not have a statistically significant influence on the severity of COVID-19 symptoms (Table 1).

**Table 1:** Variables analyzed, and corresponding *P*-value based on COVID-19 outcomes; level 0: nonhospitalized and level 1 and 2: hospitalized

Variable(s)	COVID-19 outcomes 0	COVID-19 outcomes 1 and 2	*P*
Sex			
Female	890	14	0.33^a^
Male	236	1	
Age			
1	883	10	0.35^a^
2	253	5	
BMI			
<30	852	4	0.17^a^
>30	33	1	
Confirmed case	49	11	**0.0001**
COVID-19			
symptoms	135	6	0.057
Fever	88	0	0.163
Loss of smell/taste	11	3	0.003
Pneumonia			
Required ventilation	0	4	**0.001**
Current DMT			
No	101	0	0.38^a^
Yes	867	13	
Comorbidities	319	9	**0.017** ^a^
Cardiovascular	13	0	0.81
Immunodeficiency	27	2	**0.03**
Malignancy	11	1	0.21
Neuromuscular	24	1	**0.046**
Hypertension	49	4	**0.005**
CKD	3	1	**< 0.0001**
Lung disease	31	1	0.47
DM	16	1	0.28
Duration of the			
disease (years)			0.357^a^
0–10	444	5	
11–20	522	3	
21–30	47	1	
31–40	14	0	
>40	2	0	
MS type			
RR	894	12	0.73
Progressive	102	2	
Other	130	1	
DMT			
Low efficacy	216	5	0.59^a^
Medium efficacy	293	3	
High-efficacy	175	2	
other	183	2	

Bolded values represent statistical significance.

a Fisher exact test.

CKD, chronic kidney disease; DM, diabetes mellitus; DMT, disease-modifying therapy; MS, multiple sclerosis; RR, relapsing-remitting.

## Discussion

The intricate relationship between COVID-19 and pwMS has emerged as a compelling area of investigation during the global pandemic. Our study, based on the expansive dataset from the MS Global Data Sharing Initiative, provided by PhysioNet, provides vital information on this intersection. A key finding from our study is the significant role of comorbidities in determining the outcomes of COVID-19 in pwMS. Specifically, neuromuscular disorders, hypertension, CKD, and immunodeficiencies emerged as critical factors. Neuromuscular disorders can compromise respiratory function, leading to an increased susceptibility to respiratory complications from COVID-19, a trend observed in the wider population^12^. Hypertension has been consistently identified as a risk factor for severe outcomes of COVID-19, potentially due to associated cardiovascular complications that can be exacerbated by the virus^13^. Immunodeficiencies, whether inherent or acquired, can attenuate the body’s ability to mount an effective response against SARS-CoV-2, leading to extended and more severe disease courses^14^. Within our cohort we did not observe any statistical correlation between specific symptoms related to COVID-19 and worst outcomes. However, as mentioned in the methods section a significant portion of the dataset was missing symptomatology.

Our study offers a fresh perspective on the relationship between DMT and the severity of COVID-19 in pwMS. Contrary to the initial fears of the MS community, our analysis suggests that high-efficacy DMTs do not exacerbate the severity of COVID-19. This observation is consistent with several other studies and alleviates concerns about the use of DMT in MS patients during the ongoing pandemic^15,16^. A prospective analysis of 40 MS patients also showed that the type of DMT did not significantly influence the severity of COVID-19^17^. It underscores the notion that while DMTs modify the immune response, they do not necessarily increase susceptibility to severe infections, including those caused by SARS-CoV-2. One hypothesis suggests that immunomodulators may be inversely correlated with COVID-19-related mortality due to decreased inflammation and cytokine storm; however, more studies are needed to validate this^18^. Similarly, prior use of steroids as a treatment was not correlated with worse outcomes.

The type of MS, whether it was relapsing-remitting or primary progressive, did not emerge as a significant determinant of the severity of COVID-19 in our cohort. This suggests that the inherent pathophysiology of the specific MS type may be less influential in determining the outcomes of COVID-19 compared to other factors such as age, duration of the disease, or associated comorbidities. Despite this, few studies have shown otherwise. Studies have demonstrated that people with progressive MS are more likely to be hospitalized than those with RRMS^19,20^. Furthermore, a Swedish registry cohort analysis found that those with progressive MS are more likely to have a severe infection^21^. *Januel et al*.^22^ also demonstrated that individuals with primary progressive MS are more likely to develop a severe COVID-19 infection, and anti-CD20 therapy may also be associated with the worst outcomes in those with RRMS. However, it is worth noting that the role of MS type in infectious disease outcomes remains a debated topic, warranting further investigation. These differences observed between our study and others might include variations in the study population, differences in methodologies, sample sizes, or temporal factors like the phase of the pandemic when the research was conducted.

Our study, although comprehensive, has several limitations. Reliance on self-reported or clinically reported data introduces potential biases. The granularity of the dataset did not allow us to dive deeper into the severity or control of comorbid conditions, which can significantly influence the outcomes. Additionally, the dataset lacked adequate information on individual EDSS scores, and only a small proportion of the reported sample size tested positive for COVID-19. The lack of a contemporaneous non-MS control group restricts direct comparisons. The data’s temporal limitation, with the collection tool being decommissioned in early 2022, also means that the long-term implications of COVID-19 on this cohort remain an enigma. Improved symptom and severity tracking overtime may provide further insights on how they relate to pre-existing comorbidities and different class of DMTs, which could not be ascertained from this database. While our study sheds light on the immediate relationship between MS, its therapeutic management, and COVID-19, the long-term landscape remains shrouded in uncertainty. Longitudinal studies focusing on long COVID or postacute sequelae of SARS-CoV-2 infection in pwMS are crucial. One such analysis in pwMS after COVID-19 infection did not demonstrate an increase in long-term disease activity^23^. In addition, mechanistic studies to elucidate the underlying biology of the observed associations could not only clarify these relationships but also pave the way for therapeutic advancements in the management of viral infections in pwMS.

The study’s finding that comorbidities like neuromuscular disorders, hypertension, chronic kidney disease, and immunodeficiencies significantly worsen COVID-19 outcomes in pwMS can steer future health policies toward enhancing protective measures for this group. Policies could prioritize pwMS, especially those with identified comorbidities, for vaccinations, and tailored public health initiatives, while ensuring continuity of their DMTs during pandemics. Additionally, these insights could inform healthcare resource allocation, emphasizing the need for targeted screening and management of comorbid conditions to mitigate the risks of infectious diseases in vulnerable populations.

## Conclusions

Using a comprehensive dataset from the MS Global Data Sharing Initiative provided by PhysioNet, we were able to dissect various factors that influence the outcomes of COVID-19 in pwMS. A key takeaway from our research is the pronounced impact of comorbidities such as neuromuscular disorders, hypertension, CKD, and immunodeficiencies in determining the severity of COVID-19 outcomes in pwMS. This underscores the importance of comprehensive health assessment that extends beyond the primary disease condition to include associated comorbidities for this patient population. Type of MS, duration of the disease, and high-efficacy DMT did not significantly influence the outcomes of COVID-19. Major limitations of this study include reliance on self-reported data, lack of EDSS data, and a small proportion of the reported sample size tested positive for COVID-19. Although our study provides a robust perspective, it also opens avenues for future research in developing future healthcare policies and in exploring the mechanisms by which DMTs and different types of MS may or may not influence the severity of infectious diseases such as COVID-19.

## Ethical approval

This credentialed open-access database was obtained from PhysioNet. This original study followed the ethical guidelines and received approval from the ethics committee of Hasselt University (The Ethical Committee UHasselt, CME2020/025 AMD3) (https://doi.org/10.13026/feem-fn23).

## Consent

Written informed consent was obtained from the patient for publication and any accompanying images. A copy of the written consent is available for review by the Editor-in-Chief of this journal on request.

## Sources of funding

No internal or external funding was received for this manuscript.

## Author contribution

M.A.G.-D.: conceptualization, data curation, formal analysis, methodology, project administration, resources, software, validation, visualization, writing – original draft, and writing – review and editing; B.S.S.: conceptualization, methodology, writing – original draft, and writing – review and editing; V.K.: writing – original draft.

## Conflicts of interest disclosures

On behalf of all authors, the corresponding author states that there are no conflicts of interest.

## Research registration unique identifying number (UIN)

PhysioNet https://doi.org/10.13026/feem-fn23.

## Guarantor

Bahadar S. Srichawla.

## Data availability statement

Data is available upon reasonable request from the Editor-In-Chief.

## Provenance and peer review

Not commissioned, externally peer-reviewed.
